# Complete Genome Sequences of *Pseudomonas aeruginosa* Phages vB_PaeP_PcyII-10_P3P1 and vB_PaeM_PcyII-10_PII10A

**DOI:** 10.1128/genomeA.00916-16

**Published:** 2016-11-17

**Authors:** Christine Pourcel, Cédric Midoux, Libera Latino, Marie-Agnès Petit, Gilles Vergnaud

**Affiliations:** a Institute for Integrative Biology of the Cell (I2BC), CEA, CNRS, Université Paris-Sud, Université Paris-Saclay, Gif-sur-Yvette, France; b Micalis Institute, INRA, AgroParisTech, Unive. Paris-Saclay, Jouy-en-Josas, France

## Abstract

vB_PaeP_PcyII-10_P3P1 and vB_PaeM_PcyII-10_PII10A are *Pseudomonas aeruginosa* bacteriophages belonging, respectively, to the *Lit1virus* genus of the *Podoviridae* family and the *Pbunavirus* genus of the *Myoviridae* family. Their genomes are 72,778 bp and 65,712 bp long, containing 94 and 93 predicted open reading frames, respectively.

## GENOME ANNOUNCEMENT

Using PcyII-10, a clinical strain of *Pseudomonas aeruginosa*, as the host strain, two virulent bacteriophages were isolated in Orsay, France, in the year 2014. Phage vB_PaeP_PcyII-10_P3P1 (P3P1) was found in compost and produced large clear plaques, whereas phage vB_PaeM_PcyII-10_PII10A (PII10A) was isolated from wastewater and formed small clear plaques. PII10A and P3P1 could be propagated on nine and 10, respectively, out of 27 tested clinical strains representative of the *P. aeruginosa* diversity (1). They were not capable of forming plaques on three different mutants of the lipopolysaccharide (LPS) biosynthesis pathway (2). The morphology of phage virions was determined using transmission electron microscopy (TEM). TEM was performed at 80 kV in JEM-100B (JEOL) with use of negative contrast with 1% uranyl acetate. P3P1 showed a 74 ± 0.6-nm icosahedral capsid with a short tail, characteristic of *Podoviridae*, and PII10A showed a 70 ± 1-nm head and a 130 ± 3-nm nonflexible contractile tail characteristic of *Myoviridae*.

Phage DNA was sequenced in an Illumina MiSeq 300-bp paired-end run with a 900-bp insert library produced by mechanical shearing at the IMAGIF Sequencing facility. Quality-controlled trimmed reads were assembled, using Geneious R9, to a single linear contig at mean coverages of 2,932-fold for P3P1 and 2,447-fold for PII10A. The P3P1 genome was 72,778 bp long, with a G+C content of 56%. A 671-bp direct terminal region was observed, and a homopolymer of Cs was found at position 71026 in an intergenic region upstream of open reading frame (ORF)86. Heterogeneity in the number of Cs reduced the sequencing quality at this position. The PII10A genome was 65,712 bp long with a GC content of 55.5%. Automatic ORF detection and annotation were performed with RAST, and the obtained annotations were confronted to function predictions made using Phagonaute, a website allowing the display of distant protein homologies detected with HHsearch (3), within a genomic context (4). Analysis with Phagonaute (probability threshold of 90%, followed by manual inspection) had contrasting effects on the two genomes. For PII10A, the number of annotated ORFs increased from 13 to 36: 12 ORFs were correctly annotated, one ORF was moved back to “hypothetical protein,” and 24 ORFs more received predictive annotations. In contrast, for phage P3P1, the number of annotated ORFs decreased from 38 to 32, which we interpret as RAST overannotations: 25 annotations were conserved, nine ORFs were moved back to “hypothetical protein,” and four ORFs received a different annotation. Only three additional genes (terminase small subunit, HNH endonuclease, and tail fiber protein) were annotated.

The overall P3P1 gene organization was that of phages vB_PaeP_C2-10_Ab09 and Lit1, with the long RNA polymerase characteristic of N4-like viruses (5). PII10A showed a gene organization similar to that of PB1-like phages and was most closely related to vB_PaeM_PAO1_Ab27 (1, 6). The highest level of differences was seen in the tail length tape-measure protein (ORF15), which was highly similar to that of phage NH-4 (7) and phages KTN6 and KT28 (8).

### Accession number(s).

The complete sequences of *P. aeruginosa* phages P3P1 and PII10A have been deposited in the European Nucleotide Archive (ENA) under the accession numbers LT594787 and LT594786, respectively, within BioProject PRJEB14592, together with the raw sequence reads. The versions described in this paper are the second versions.
